# Adalimumab and sulfasalazine in alleviating sacroiliac and aortic inflammation detected in PET/CT in patients with axial spondyloarthritis: PETSPA

**DOI:** 10.1002/iid3.552

**Published:** 2021-11-09

**Authors:** Juha‐Pekka Kaijasilta, Anne M. Kerola, Riitta Tuompo, Heikki Relas, Antti Loimaala, Hannu Koivu, Jukka Schildt, Tuomas Kerola, Kari Eklund, Markku J. Kauppi, Tuomo V. M. Nieminen

**Affiliations:** ^1^ Faculty of Medicine University of Helsinki Helsinki Finland; ^2^ Department of Internal Medicine Päijät‐Häme Central Hospital Lahti Finland; ^3^ Department of Rheumatology, Inflammation Center Helsinki University Hospital Helsinki Finland; ^4^ Medical Imaging Center Helsinki University Hospital Helsinki Finland; ^5^ Department of Nuclear Medicine Päijät‐Häme Central Hospital Lahti Finland; ^6^ Orton Hospital Helsinki Finland; ^7^ Translational Immunology Program (TRIMM), Faculty of Medicine University of Helsinki Helsinki Finland; ^8^ Department of Internal Medicine Helsinki University Hospital Helsinki Finland; ^9^ Department of Internal Medicine South Karelia Central Hospital Lappeenranta Finland; ^10^ Päijät‐Häme Joint Authority for Health and Wellbeing Lahti Finland

**Keywords:** adalimumab, axial spondyloarthritis, inflammation, positron emission tomography/computed tomography, sulfasalazine

## Abstract

**Aim:**

Inflammatory signals in the sacroiliac (SI) joints and the aorta of patients with axial spondyloarthritis (axSpA) were graded by positron emission tomography/computed tomography (PET/CT) imaging before and after treatment with sulfasalazine (SSZ) or adalimumab (ADA).

**Methods:**

Patients with axSpA, Bath Ankylosing Spondylitis Disease Activity Index (BASDAI) ≥ 4, were recruited. Disease‐modifying antirheumatic drug‐naïve patients started SSZ for 12 weeks, whereas those with prestudy treatment with or contraindication to SSZ commenced ADA for 16 weeks. In addition, those patients in the SSZ group with insufficient response commenced ADA for 16 weeks. 18F‐fluorodeoxyglucose PET/CT was performed after inclusion and after treatment with SSZ and ADA. Maximum standardized uptake value (SUVmax) was assessed for the aorta and the SI joints, and maximal target‐to‐blood‐pool ratio (TBRmax) only for the aorta.

**Results:**

Among five SSZ patients, mean ± SD BASDAI was 4.7 ± 1.6 before and 3.5 ± 1.4 after treatment (*p* = .101). In 13 ADA patients, the BASDAI decreased from 5.4 ± 1.6 to 2.8 ± 2.2 (*p* < .001). Among the SSZ patients, SUVmax in SI joints decreased from 2.35 ± 0.55 to 1.51 ± 0.22 (−35.8%, *p* = .029). Aortic TBRmax decreased from 1.59 ± 0.43 to 1.26 ± 0.26 (−33.2%, *p* = .087). In the ADA patients, SUVmax in the SI joints was 1.92 ± 0.65 before and 1.88 ± 0.54 after treatment (−1.8%, *p* = .808) and TBRmax in the aorta 1.50 ± 0.60 before and 1.40 ± 0.26 after treatment (−6.7%, *p* = .485).

**Conclusions:**

Our small open‐label study showed that SSZ may reduce PET‐CT‐detectable inflammation in the SI joints, with a trend towards a reduction in the aorta.

## Graphical abstract

We launched this small open‐label study to test the hypothesis that axial spondyloarthritis (axSpA) induces positron emission tomography (PET)‐detectable inflammation in the sacroiliac (SI) joints and cardiovascular tissues and that the effects of treatment with sulfasalazine and adalimumab (ADA) for axSpA can be visualized and confirmed by PET. Our results imply that sulfasalazine may reduce PET‐computed tomography‐detectable inflammation in the SI joints, with a trend towards a reduction in the aorta. In the ADA group, however, we did not observe any change in the PET signals in the SI joints nor in the aorta.

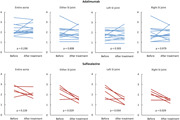

## 1 INTRODUCTION

Axial spondyloarthritis (axSpA) is an inflammatory rheumatic disease mainly affecting joints in the spine and the sacroiliac (SI) joints. The presence of inflammatory back pain in a young adult usually raises a suspicion of the disease. According to the modified New York criteria and the more recent Assessment in SpondyloArthritis International Society (ASAS) classification criteria, the detection of sacroiliitis is central to the diagnosis of axSpA.^1^ Conventional radiograph may be used as an initial step. Computed tomography (CT) is superior in detecting structural damage. Active inflammation is not visible in either modality.^2^ Magnetic resonance imaging (MRI) is considered to be the most sensitive modality in clinical practice for detecting inflammation in the SI joints and the spine in axSpA.

Positron emission tomography (PET)/CT is a nuclear imaging technique that produces a three‐dimensional (3D) image of functional processes in the body. The tracers used in the examination accumulate in tissues with metabolic activity. For example, the most commonly used tracer, 18F‐fluorodeoxyglucose (18F‐FDG), gravitates towards regions with glucose uptake. The quality of the 3D imaging is enhanced with the aid of a CT scan. The PET/CT technology is increasingly used in rheumatologic and cardiologic indications, as it is the most sensitive method to detect inflammatory foci in clinical practice. The imaging of atherosclerotic inflammation is highly reproducible with 18F‐FDG PET.^3^ According to the small patient series, 18F‐fluoride PET/CT may be useful in detecting active sacroiliitis.^4^, ^5^ To our knowledge, only one patient series (*n* = 3) has documented the effect of anti‐tumor necrosis factor (TNF) treatment on inflammatory lesions in the spine with 18F‐FDG PET scans in ankylosing spondylitis.^6^ The first‐line treatment of axSpA comprises nonpharmacological treatment (such as exercise and physical therapy) and nonsteroidal anti‐inflammatory drugs. With regard to biologic disease‐modifying antirheumatic drugs (DMARDs), TNF‐α inhibitors are highly effective in alleviating symptoms and decreasing C‐reactive protein (CRP) levels and MRI‐detectable inflammation. Conventional synthetic DMARDs can also be considered, especially in peripherally active diseases.^7^

AxSpA is associated with an approximately 1.5–2‐fold risk of atherosclerotic cardiovascular disease (CVD).^8^, ^9^ In addition to traditional CVD risk factors, the central link from rheumatic diseases to accelerated atherosclerosis in systemic inflammation. Treating inflammatory joint diseases effectively into remission with antirheumatic medication is considered to reduce excess CVD risk through the suppression of inflammation.^10^ Also, clinical aortitis is a well‐known, although rare, complication of axSpA.^11^ Inflammation in the aortic wall due to either atherosclerosis or vasculitis can be evaluated with PET/CT.^12^ This rationale supports the use of PET/CT to detect inflammation in the aortae of patients with axSpA.

As the PET data on axSpA patients is scarce, we launched a study to test the hypothesis that axSpA induces PET‐detectable inflammation in the SI joints and the aorta, and that the effects of treatment with sulfasalazine (SSZ) and adalimumab (ADA), separately, can be visualized and confirmed by PET, the gold standard for detecting inflammatory foci in clinical practice.

## 2 METHODS

### 2.1 Study design

**Figure 1:**
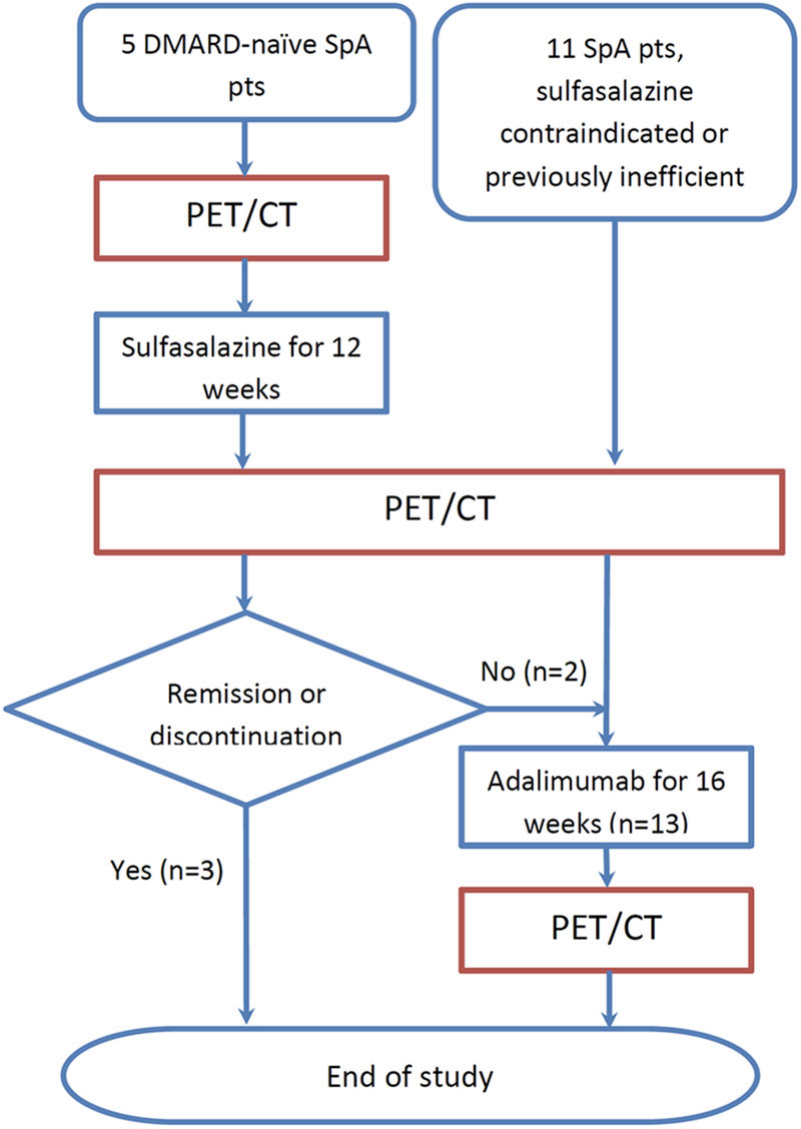
Study flow for the 16 patients and 18 treatment periods. DMARD, disease‐modifying antirheumatic drug; PET/CT, positron emission tomography/computed tomography; pts, patients; SpA, spondyloarthritis

The PETSPA study is a nonrandomized uncontrolled clinical trial to study the effects of two types of antirheumatic treatments, SSZ, and ADA, on PET/CT‐detectable inflammation in the SI joints and aortae of patients with axSpA. Two types of patients were recruited in the study (Figure 1).

1. DMARD‐naïve patients with no contraindications to SSZ treatment were started on SSZ for 12 weeks.

2. Those with prestudy treatment with SSZ with insufficient response or contraindication to SSZ commenced ADA 40 mg s.c. every 2 weeks for 16 weeks.

In addition, the patients in the SSZ group with insufficient response to SSZ commenced ADA for 16 weeks. Patients were scanned with PET/CT before and after treatment periods with SSZ and/or ADA.

Here, we report the results of the pilot phase, which was planned to determine whether it is plausible to detect significant changes in the PET signals of the aorta and the SI joints of patients with axSpA. The pilot phase revealed significant changes in the SI joints in the SSZ group. The signals in the SI joints for the ADA group and the signals in the aorta for both groups clearly suggested that no essentially additional information would be detected even with 60 patients, which was the planned maximum number of recruited patients (20 patients with SSZ and 40 patients with ADA). In addition, the recruitment was deemed too slow for the study to continue. For these reasons, recruitment was terminated after the pilot phase.

The Ethics Committee of the Hospital District of Helsinki and Uusimaa approved the study protocol, and all patients gave informed consent as stipulated in the Declaration of Helsinki.

### 2.2 Patients

Twenty axSpA patients with sacroiliitis, as detected by either MRI or X‐ray, and active inflammation indicated by an elevated Bath Ankylosing Spondylitis Disease Activity Index (BASDAI) (4.0 or higher) were recruited from the rheumatology clinics of Helsinki University Central Hospital (*n* = 14) and Päijät‐Häme Central Hospital (*n* = 6). The diagnosis of axSpA was based on the ASAS recommendation's imaging arm.^1^, ^13^ The exclusion criteria were as follows: psoriasis or psoriatic arthritis, inflammatory bowel disease, probable noncompliance or unwillingness to participate in the study with additional imaging protocols, expected life‐span of less than 1 year, diabetes (to improve the PET imaging quality), pregnancy, age <18 years or >75 years, as well as methotrexate used within the previous 6 months and a biologic medicine used within the previous 6 months. The practice guidelines were approved by each center's institutional review board and ethics committee.

One patient withdrew the informed consent, one did not arrive for the scans, and one suspected she was pregnant before the PET/CT scan. PET/CT scans before and after treatment with antirheumatic drugs were performed on 17 patients. One patient was scanned with two different types of PET equipment, which precluded comparison. Thus, 16 patients and a combined 18 treatment periods with either SSZ and/or ADA were included in the analysis.

### 2.3 The rationale for using SSZ as the first‐line DMARD therapy

The Finnish regulations for drug purchase reimbursement in the treatment of axSpA are strict and require that at least one conventional DMARD needs to fails to alleviate symptoms or has to cause significant side effects before the reimbursement of biologic DMARDs, including TNF‐α inhibitors, is considered. A study based on a Finnish nationwide register of special reimbursements for medication costs in axSpA demonstrated that SSZ monotherapy was by far the most common first antirheumatic treatment (87%), followed by methotrexate monotherapy (9%). A combination of two or more DMARDs was used rarely (2%). Only a remarkably small proportion of the patients (0.3%) had a biological drug (ADA or etanercept) commenced as the first‐line antirheumatic drug.^14^ Patients undergoing ADA treatment, thus, had a history of treatment failure with at least one conventional DMARD, mostly SSZ. Typically, SSZ is started with 500 mg twice a day and titrated to up to 2000–3000 mg per day.

### 2.4 Primary endpoint

The primary endpoint was a decrease in PET signal levels both in the aorta (maximal target‐to‐blood‐pool ratio [TBRmax] in the whole aorta) and the SI joints (maximum standardized uptake value [SUVmax]) after treatment with either SSZ or ADA. The variables of most interest reflect intraindividual changes.

### 2.5 Study flow

All the patients with axSpA were PET/CT‐scanned after inclusion in the study (Figure 1). Immediately thereafter, the DMARD‐naïve patients (*n* = 5) were started on SSZ monotherapy. ADA was commenced for those without clinical improvement (*n* = 2) at 12 weeks. These two patients were scanned with PET/CT for the third and final time after 16 weeks on ADA. For the patients who had failed on conventional DMARDs before inclusion, ADA 40 mg s.c. every 2 weeks was commenced (*n* = 11), and the PET/CT scan was repeated after 16 weeks.

Symptoms, disease activity (BASDAI), possible adverse reactions, medications, clinical rheumatologic findings, pulse rate, and blood pressure were recorded at each outpatient visit. Three months after the initiation of treatment, the free ADA concentration was measured using an enzyme‐linked immunosorbent assay, and if the value was below 2.0, antibodies were also measured. Human antihuman antibodies against ADA were quantified using a validated radioimmunoassay method.

### 2.6 Imaging

The PET‐CT scan was carried out with an 18F‐FDG tracer (3MBq/kg), with a total dose of radiation of 8 mSv (including the dose of radiation from the CT). The scanners used were Siemens Biograph Horizon and Siemens Biograph mCT (Siemens Healthineers). The patients underwent a CT and the PET scan 60 min after 18F‐FDG administration. Emission images were acquired for 3 min per bed position or 1.3 mm/s with motion flow. Ordered subset expectation maximization reconstruction method with 10 subsets, 5 iterations, and a matrix size of 180 × 180 was used, 4 mm Gaussian filter (time‐of‐flight and point spread function). The PET‐CT scan covered the body from the jugulum to the SI joints. This allowed the analysis of the aortic signal from the aortic root to the abdominal aorta, as well as the central spondyloarthritis loci, that is, the SI joints and most of the spine. The image analyses were carried out with Siemens SyngoVia (Siemens Healthineers) and HERMES HybridViewer. The most intense voxel activity within the regions of interest (SUVmax) was measured. TBRmax was assessed for the aorta by dividing SUVmax with the venous blood pool SUV. TBR has been widely used in publications regarding vascular inflammation. SUVmax was chosen for the SI joints because it is the most common quantitative method to evaluate the intensity of inflammation in other tissues.

### 2.7 Statistics

Changes due to treatments were compared with the paired *t*‐test. The statistical analyses were performed with SPSS Statistics for Windows, version 24 (IBM Corp.). A *p*‐value of <.05 was considered statistically significant.

## 3 RESULTS

There were eight men and five women in the ADA group and three men and two women in the SSZ group. The median age was 37 (range, 22–50) years in the ADA group and 29 (range, 23–55) years in the SSZ group. Eighty‐two percent of the patients were HLA‐B27 positive. Forty‐four percent had definite radiographic sacroiliitis according to the modified New York criteria, thus defined as radiographic axSpA (60% of the SSZ group and 38% of the ADA group), and the rest had nonradiographic axSpA. There were no significant comorbidities in either group.

In the group receiving ADA, the mean BASDAI decreased from 5.4 ± 1.6 to 2.8 ± 2.2 (*p* = .0001). Among those who used SSZ, BASDAI was 4.7 ± 1.6 before and 3.5 ± 1.4 after the treatment (*p* = .10). Clinical remission (defined as a >50% decrease in the BASDAI) was achieved in 62% in the ADA group and in 20% in the SSZ group. Further baseline characteristics and clinical responses are shown in Table 1.

**Table 1:** Maximal target‐to‐blood‐pool ratio (TBRmax) in the aorta and the most intense voxel activity within the regions of interest (maximal standardized uptake value [SUVmax]) in the aorta and the sacroiliac (SI) joints in consecutive PET scans in both the adalimumab group (*n* = 13) and the sulfasalazine group (*n* = 5)

	Adalimumab								Sulfasalazine							
	Before		After		Change				Before		After		Change			
	Mean	SD	Mean	SD	%	*df*	*t*	*p* (*t*‐test)	Mean	SD	Mean	SD	%	*df*	*t*	*p* (*t*‐test)
Aortic TBRmax	1.50	0.60	1.40	0.26	−6.7	12	0.72	.485	1.59	0.43	1.26	0.26	−33.2	4	2.26	.087
Aortic SUVmax	2.18	0.54	2.33	0.54	6.8	12	−1.24	.238	2.44	0.46	2.07	0.43	−15.0	4	−1.18	.226
SI joints SUVmax	1.92	0.65	1.88	0.54	−1.8	12	0.25	.808	2.35	0.55	1.51	0.22	−35.8	4	3.33	.029
Left SI joint SUVmax	1.74	0.47	1.81	0.53	4.3	12	−0.69	.504	2.22	0.61	1.37	0.34	−38.4	4	3.16	.034
Right SI joint SUVmax	1.83	0.69	1.84	0.55	0.3	12	−0.04	.971	2.08	0.40	1.44	0.17	−30.5	4	3.33	.029
Blood glucose	5.8	0.7	5.8	0.5					6.0	0.9	5.9	0.8				
C‐reactive protein	18.7	16.6	2.3	3.0	−87.5	12	3.84	.002	9.2	10.9	19.7	25.6	114.1	4	−1.47	.217
ESR	19.1	16.2	5.5	4.5	−71.5	10	2.91	.016	18.8	12.0	27.3	25.6	45.3	3	−0.79	.487
BASDAI	5.4	1.6	2.8	2.2	−47.9	12	5.63	<.001	4.7	1.6	3.5	1.4	−24.0	4	2.12	.101

*Note*: Clinical characteristics are shown before and after treatment. Clinical response is shown with inflammation markers and symptom scores.

Abbreviations: BASDAI, Bath Ankylosing Spondylitis Disease Activity Index; ESR, erythrocyte sedimentation rate; PET, positron emission tomography.

**Figure 2:**
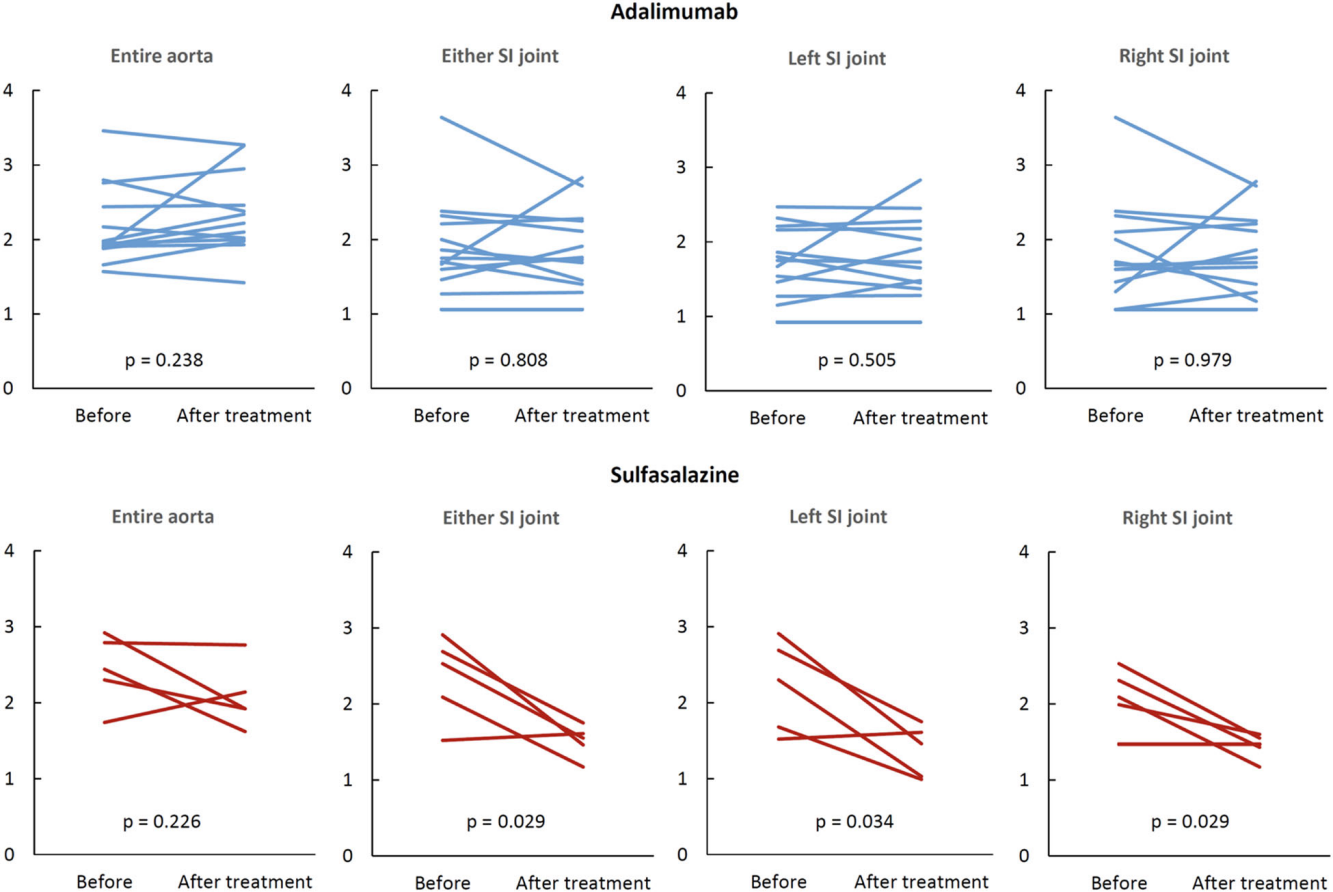
PET activity in the entire aorta (SUVmax) as well as the sacroiliac (SI) joints (SUVmax) for each patient within the adalimumab (blue lines) and sulfasalazine (red) groups. The *p* values are from paired *t*‐tests. PET, positron emission tomography; SUVmax, maximum standardized uptake value

The levels of PET signals for both groups are given in Table 1. The values for each patient are illustrated in Figure 2. Among the SSZ group, the SUVmax in the SI joints declined markedly from 2.35 ± 0.55 to 1.51 ± 0.22 (−35.8%, *p* = .022), with a decreasing trend for aortic TBRmax from 1.59 ± 0.46 to 1.26 ± 0.43 (−33.2%, *p* = .087). The PET activity did not change markedly in the patients receiving ADA.

Seven out of the 13 (54%) ADA patients had received SSZ treatment before the baseline PET. Compared to the SSZ group, the baseline average aortic PET signal in the ADA group was similar (TBRmax 1.50 vs. 1.59), but that of the SI joints was somewhat lower in the ADA group (SUVmax 1.97 vs. 2.35).

The baseline SUVmax measured from the SI joints was 2.17 ± 0.86 in radiographic axSpA as compared to the 1.84 ± 0.31 in nonradiographic axSpA (*p* = .120). Among patients with radiographic axSpA, the SUVmax decreased to 1.80 ± 0.66 (−17.4%, *p* = .189) when both drugs where included. In patients with nonradiographic axSpA, the SUVmax in the SI joints was 1.77 ± 0.26 after treatment (4.1%, *p* = .403).

The clinical response to ADA was not associated with PET/CT results. In patients with a good clinical response (*n* = 8, >50% reduction in BASDAI), the SUVmax in the SI joints was 1.84 ± 0.39 before and 1.91 ± 0.49 after treatment (*p* = .72). In patients with a poor clinical response (*n* = 5, <50% decrease in BASDAI), the SUVmax in the SI joints was 2.06 ± 0.99 before and 1.83 ± 0.66 after treatment (*p* = .29).

## 4 DISCUSSION

Our study demonstrates, for the first time, that SSZ may reduce inflammation in the SI joints, as detected by reduced 18F‐FDG uptake in PET/CT. There was also a trend towards the alleviation of inflammation in the aorta. PET activity decreased as much as by roughly a third in both tissues during SSZ treatment. The interpretation of the results is limited by the small sample size. In addition, the changes in the PET signals of the SI joints were not consistent with the treatment response.

PET/CT has rarely been studied in the context of axSpA. The 18F‐fluoride tracer has been used in some of the studies. In contrast to 18F‐FDG, 18F‐fluoride uptake might not only be a consequence of inflammation but also an indicator of postinflammatory repair osteoproliferation.^5^ Quantitative 18F‐fluoride PET/CT has been used to diagnose sacroiliitis in a small study on 15 patients, and PET/CT was a relatively sensitive (80%) and specific (77%) modality with conventional radiographs as the reference.^5^ The reduction in signal intensity measured with 18F‐fluoride PET/CT correlated with clinical disease activity, as measured with the ankylosing spondylitis disease activity score in axSpA patients (*n* = 29).^15^ Vijayant et al.^16^ used PET/CT with 18F‐FDG in a small study including seven patients with ankylosing spondylitis and showed FDG hypermetabolism in the SI joints bilaterally, as well as significant tendon and muscular uptake corresponding to the symptomatic joints. In another study (21 patients with SpA, 16 with polymyalgia rheumatica, and 16 with rheumatoid arthritis), FDG uptake in the SI joints was significantly higher in SpA than in other rheumatic diseases.^17^

Studies examining the response to antirheumatic treatment in axSpA with PET/CT are scarce. To the best of our knowledge, only in one case series of three patients with ankylosing spondylitis, PET/CT showed a reduction in FDG uptake in spondylodiscitis lesions during treatment with TNF inhibitors.^6^ In contrast, the treatment response to SSZ and the TNF‐α inhibitor etanercept has been evaluated with MRI: in the ESTHER study, etanercept reduced active inflammatory lesions detected in MRI more than SSZ, and this effect correlated with a good clinical response in the etanercept group.^18^

In our study, ADA and SSZ both seemed to reduce clinical symptoms, even though SSZ did not reach statistical significance, probably due to the small number of patients. This is in line with the broad evidence of the effectiveness of ADA in decreasing disease activity and enhancing functional capacity in axSpA. PET/CT, however, could not show any reduction in FDG uptake in the SI joints nor the aorta in the ADA group. This may be partly due to the fact that ADA was used as the second‐line treatment, when the inflammation had already been reduced close to the limit detectable by the method. More than half of the ADA patients had received SSZ treatment immediately before the baseline PET scan. However, the patients still had an active enough disease to be recruited in the study.

According to the latest Cochrane systematic review, the information available about the effectiveness of SSZ in treating axSpA is limited, as no large‐scale randomized controlled trials have been performed.^19^ SSZ seems to ease spinal stiffness and reduce the erythrocyte sedimentation rate and CRP levels.^19^, ^20^, ^21^ SSZ may be effective in axSpA, especially in younger patients and short disease duration.^20^ Our study further implies SSZ's potential to reduce inflammation, but our findings need to be confirmed in larger studies, preferably with a randomized design.

To the best of our knowledge, no study to date has evaluated PET/CT‐detectable inflammatory signals in the aorta among axSpA patients. In our study, TBRmax values in the aortae of the 16 relatively young axSpA patients were generally low and did not suggest aortic inflammation due to atherosclerosis or subclinical aortitis. We were not able to show a significant reduction in aortic PET signals after antirheumatic treatment in either the SSZ or the ADA group. However, there was a trend towards a reduction in aortic TBR in the SSZ group, which lacked statistical significance, however, perhaps due to a lack of power. Clinically active aortitis is rare in axSpA and was not diagnosed in either treatment group.

PET/CT with 18F‐FDG has been used in other rheumatic diseases to detect vascular inflammation. Psoriasis has been shown to increase TBR values in the aorta when compared to age‐ and body mass index‐matched controls.^22^ In rheumatoid arthritis, TNF‐α therapy has been demonstrated to reduce aortic inflammation, as shown by TBRmax values in consecutive imaging scans (from 2.02 ± 0.22 to 1.90 ± 0.29, *p* = .03).^23^

We conducted a small open‐label study, and we did not have healthy controls, which are important limitations. It was considered unethical to undertake a double‐blind randomized trial on axSpA patients with severely symptomatic disease. 18F‐FDG was chosen as a tracer to evaluate not only bone lesions but also inflammation in the aorta. 18F‐fluoride has been shown to be more sensitive in bone lesions.^24^ The small number of patients might play a role in the fact that SSZ treatment performed better than expected in reducing inflammatory signals in PET/CT. AxSpA may have a fluctuating disease course, and the decrease in the inflammation measured by means of PET/CT may, therefore, be partly due to the natural course of the disease rather than the drug therapy. Despite the low number of patients in the two separate drug arms, we aimed to analyze the effects of SSZ and ADA on PET/CT detectable inflammation separately as described in the protocol, because this was an open‐label study and clinical situations where SSZ and ADA are initiated differ (ADA is generally initiated only if treatment with SSZ fails).

## 5 CONCLUSION

Our small open‐label study showed that SSZ may reduce inflammation as detected by 18F‐FDG PET/CT in the SI joints. There was also a trend towards a reduction in inflammation in the aorta. In contrast, disease activity was significantly reduced only in the ADA group, although a trend towards a reduction in BASDAI was also seen in the SSZ group. Further research is warranted on the usefulness of PET/CT in the evaluation of the activity of sacroiliitis and vascular inflammation in the context of rheumatic diseases.

## CONFLICT OF INTERESTS

Anne M. Kerola has received speaker fees from Boehringer‐Ingelheim and Sanofi, has attended advisory boards of Pfizer, Gilead, and Boehringer‐Ingelheim, and received congress sponsorship from Pfizer, Celgene, UCB Pharma, Mylan, and Roche. Riitta Tuompo has received speaker fees from Mylan, and congress sponsorship from Roche and Novartis. Other authors declare that there are no conflict of interests.

## AUTHOR CONTRIBUTIONS

Juha‐Pekka Kaijasilta participated in the conception of the work, the interpretation of data for the work, and the critical revision of the manuscript for important intellectual content. Anne M. Kerola and Tuomo V. M. Nieminen participated in the conception of the work, analysis, interpretation of data for the work, and the critical revision of the manuscript for important intellectual content. Riitta Tuompo, Heikki Relas, Antti Loimaala, Hannu Koivu, Jukka Schildt, Tuomas Kerola, Kari Eklund, and Markku J. Kauppi participated in the acquisition of the data analysis, interpretation of data, and in the critical revision of the manuscript for important intellectual content.

## ETHICS STATEMENT

The practice guidelines were approved by each center's institutional review board and Finnish national ethics committee (Reference Number 75/23/03/01/2015). All patients enrolled in the study gave signed informed consent.

## Data Availability

The data that support the findings of this study are available on request from the corresponding author. The data are not publicly available due to privacy or ethical restrictions.
